# Fatal Poisoning from Carbolic Acid

**Published:** 1874-01

**Authors:** 


### FATAL POISONING FROM CARBOLIC ACID.

A woman in Louisville, Ky., had a mammary tumor excised. Four days afterward her nurse gave her a tablespoonful of carbolic acid, diluted with an ounce of water. She complained immediately of its burning, and the nurse tried to provoke vomiting, without success. In less than ten minutes she was unconscious, pupils contracted and insensible, respiration 50, pulse 150, clammy perspiration. Death in two hours.—*Am. Practitioner.*

